# Patients with post-COVID-19 condition show minor blood transcriptomic changes, with altered erythrocyte gene expression in a male subgroup

**DOI:** 10.3389/fimmu.2025.1500997

**Published:** 2025-03-21

**Authors:** Piia Karisola, Mari Kanerva, Aki Vuokko, Helena Liira, Shuyuan Wang, Kirsi Kvarnström, Mikko Varonen, Hille Suojalehto, Harri Alenius

**Affiliations:** ^1^ Human Microbiome (HUMI) Research Program, Faculty of Medicine, University of Helsinki, Helsinki, Finland; ^2^ Department of Infection Control, TYKS Turku University Hospital, The Wellbeing Services County of Southwest Finland, Turku, Finland; ^3^ Outpatient Clinic for Long-Term Effects of COVID-19, Helsinki University Central Hospital, Helsinki, Finland; ^4^ Occupational Medicine, Finnish Institute of Occupational Health, Helsinki, Finland; ^5^ Institute of Environmental Medicine (IMM), Karolinska Institutet, Stockholm, Sweden

**Keywords:** post-COVID-19 condition, SARS-CoV-2, transcriptomics, bioinformatics, erythrocyte (human)

## Abstract

**Background:**

The mechanisms underlying persistent symptoms after non-severe COVID-19 remain unclear. This study aimed to investigate transcriptomic changes in peripheral blood cells of patients with post-COVID-19 condition (PCC) and assess if distinct clinical subtypes with specific gene signatures could be identified.

**Methods:**

The cohort included 111 PCC patients from the SARS-CoV-2 Omicron variant era, with 57 recovered (Recov) and 54 having prolonged symptoms indicative of PCC. The results were compared to 63 healthy controls (Ctrl) without known SARS-CoV-2 infection. Clinical data included patient assessments, laboratory results, comorbidities, and questionnaires on quality of life and functioning. Transcriptomic analysis and cellular deconvolution methods were used on total RNA from peripheral blood mononuclear cells (PBMCs).

**Results:**

PCC patients had more comorbidities (mean 1.3) and more frequently (59%) at least one comorbidity than recovered patients (31%) and controls (24%). Overall, past COVID-19 illness or current PCC symptoms caused minimal changes in the blood cell transcriptome, with only 3–6 differentially expressed genes (DEGs) identified across comparisons. However, a subset of male PCC patients exhibited an increased fraction of deconvoluted erythroblasts and significant genome-wide gene expression changes, with 399 DEGs compared to recovered and control males. These genes were enriched in pathways related to heme metabolism and gas exchange in erythrocytes.

**Conclusions:**

Persistent symptoms in PCC are multifactorial and not directly linked to peripheral blood cell gene expression changes. However, a subgroup of male PCC patients shows distinct erythrocyte responses that may contribute to long-term symptoms.

## 1 Introduction

COVID-19 is caused by the severe acute respiratory syndrome coronavirus 2 (SARS-CoV-2), a highly infectious respiratory virus responsible for the global pandemic. COVID-19 often presents as an asymptomatic or mild to moderate respiratory infection in previously healthy individuals with symptoms including fever, cough, headache, fatigue, myalgia, diarrhea, and anosmia (1, 2). Although the successful vaccination strategy significantly decreased morbidity and mortality (3), the emergence of SARS-CoV-2 variants due to viral mutations still raises concern due to increased transmissibility and resistance to neutralization and vaccination (4). In vulnerable individuals, mild or asymptomatic disease is followed by post-acute sequelae of COVID-19 (PASC), also referred to as long-COVID (LC) or post-COVID-19 condition (PCC) (5, 6).

Months after an acute SARS-CoV-2 infection and without other medical explanations, 10%–20% of COVID-19 patients suffer from PCC symptoms, presenting fatigue or muscle weakness, sleep difficulties, and anxiety or depression, which are ultimately accompanied by significant socioeconomic consequences (7–9). Evidence suggests that age, sex, and race can influence the development and severity of PCC (10, 11). The pathophysiological mechanisms of PCC are still under debate and are suggested to include the effects of immune responses to the persistent virus, ongoing inflammation, and autoimmune responses (7). A recent study suggests that the modified structure of skeletal muscles is associated with a lower exercise capacity in PCC patients (12). In this study, physical exercise was induced postexertional malaise (PEM), leading to local and systemic metabolic disturbances, severe myopathy, and worsening of tissue infiltration of amyloid-containing deposits in skeletal muscles (12). In addition to the many physical and cognitive complaints, others have suggested dysfunction of integrative brain regions potentially associated with central catechol pathway dysregulation, with consequences on autonomic functioning and physical conditioning (8).

To investigate the relationship between host immune responses and gene expression biomarkers, we analyzed differences between two groups of participants: those who recovered from Omicron COVID-19 without persistent symptoms (Recov) and those with ongoing PCC. Their responses were compared to those of healthy controls (Ctrl) with no known history of COVID-19 infection. From these participants, we measured various physiological and functional properties, a range of clinical serological parameters, antibodies to COVID-19 surface antigen, and related them to gene expression changes of the blood immune cells. We also tested the hypothesis that patients with PCC form distinct endotypes and revealed their gene signature associated with PCC symptoms.

## 2 Methods

### 2.1 Study design and population

This study was carried out in Finland by the University of Helsinki and Helsinki University Hospital (HUS) in collaboration with the Finnish Institute of Occupational Health.

**Figure 1:**
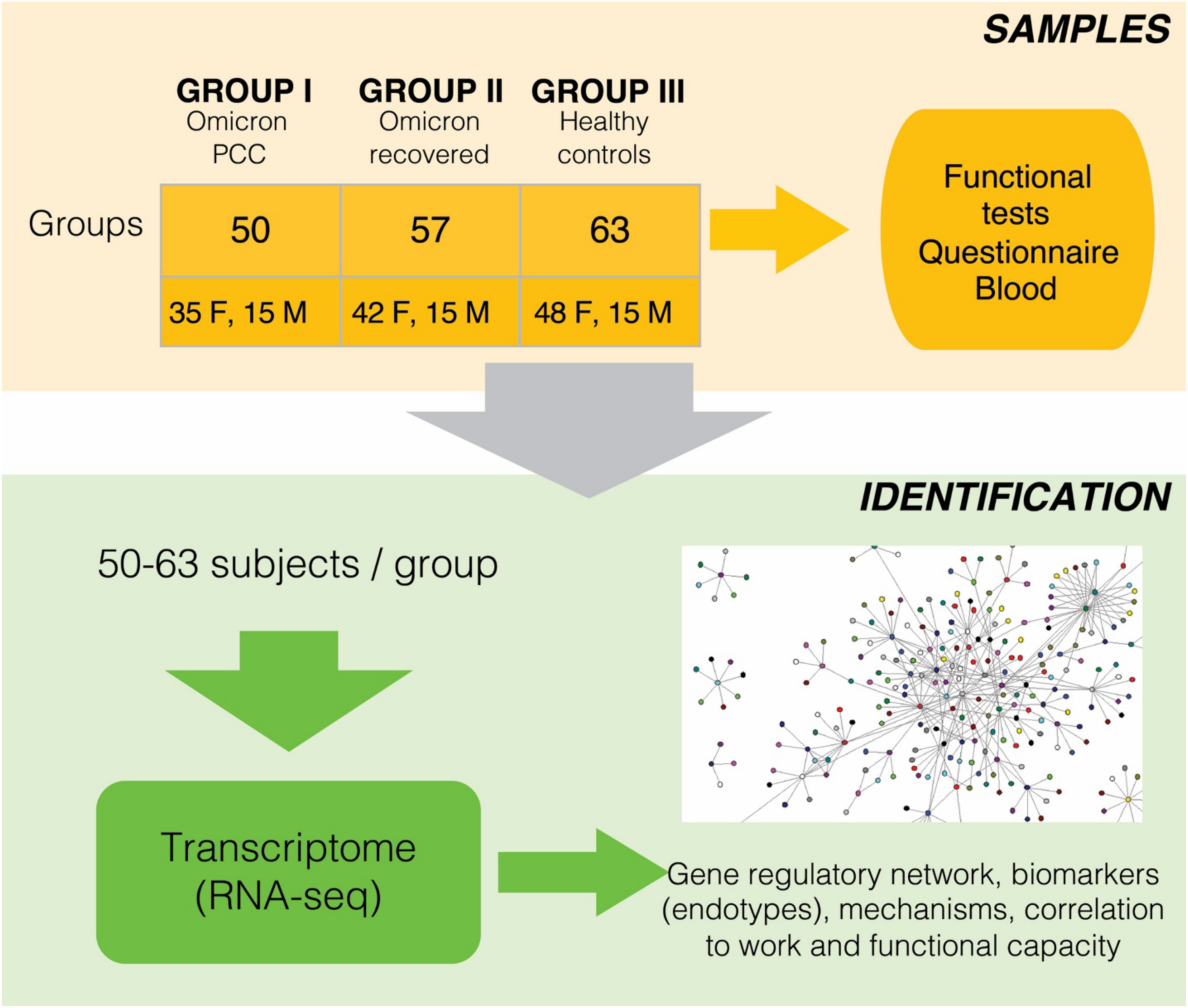
Schematic view of the study setup, sample collection and analyses. Additional info of patients and clinical parameters are in the **Tables 1**, **2**.

**Figure 1** shows the workflow of the study. All participants were between 18 and 65 years old and had been vaccinated at least twice against SARS-CoV-2. At that time, Finnish citizens were vaccinated either with the Comirnaty mRNA vaccine (Pfizer) or with the Vaxzevria adenoviral vector (Oxford–AstraZeneca) against SARS-CoV-2. Cases with PCC had previously had a laboratory-confirmed SARS-CoV-2 infection (positive nucleic acid amplification test) during 2022 and had been referred to a specialized long COVID policlinic (LCP) at HUS, where they were recruited. We used a definition of PCC as the continuation or development of new symptoms 3 months after the initial SARS-CoV-2 infection, with these symptoms lasting for at least 2 months with no other explanation (13).

Participants in the SARS-CoV-2 recovered (Recov) cohort also had a laboratory or at-home over-the-counter diagnostic test confirming SARS-CoV-2 infection in 2022 and had recovered normally. They were recruited from another outpatient COVID study cohort at HUS (14), social media advertisements, and via personal contacts of HUS Clinic for Long-Term Effects of COVID-19 staff. Participants with no known SARS-CoV-2 in history based on their own information (control group; Ctrl) were recruited by social media advertisements and via personal contacts of HUS Clinic for Long-Term Effects of COVID-19 staff. Among these Recov and Ctrl volunteers, persons with diseases markedly affecting the immune system were excluded. In these groups we recruited persons who matched the PCC patients in age (age groups 18–45 years and 46 years or over) and gender.

### 2.2 Ethical considerations

The study was approved by the HUS research ethics committee board, ID HUS/1493/2021, and the final amendments to the study protocol were approved on 13.9.2023. All eligible individuals received both oral and written information about the study protocol and provided written informed consent on inclusion (15).

### 2.3 Clinical data collection

Participant data were entered in electronic case report forms and subsequently stored on a secure research server. All cases were assessed at the polyclinic to confirm or rule out PCC symptoms by interview. They also completed questionnaires on lifestyle factors such as smoking, as well as quality of life and functioning using a VAS scale of 0–10 [see details in Virrantaus et al. (15)]. Physical function testing, supervised by the clinic’s physiotherapist, included assessments of dominant hand grip strength (HGST) in kilograms (kg) using a Jamar/Saehan dynamometer (16) and a 6-min walking test (6MWT) in meters (m) (17).

All patients provided blood samples. Comorbidities and medication were collected electronically from tertiary healthcare data by ICD-10 codes within the last 5 years from the HUS Data Lake on the HUS Academic platform and by ATC codes from medication lists at the time of the polyclinic visit. The data covered were collected from the tertiary care center (HUS) and from primary care consultations within the HUS acute departments. However, it was not possible to include data from other primary healthcare sources (public or private) or occupational healthcare data.

### 2.4 Blood sampling

The venous blood samples (7 ml each) were collected into PAX blood tubes between 1 February and 31 June 2023 (Norgen Thorol, ON, Canada). The tubes were incubated at RT overnight and transferred to –80°C for storage on the following day. Sera were stored at –80°C.

### 2.5 SARS-Cov-2 antibody luminex

The serum samples were diluted 1:100 into the Bio-Plex sample diluent, and 50 μl of the dilution was pipetted onto the plate. SARS-CoV-2 virus antigens of nucleocapsid (N), receptor binding domain (RBD), and spike proteins 1-2 were analyzed by Bio-Plex Pro Human IgG 4-plex panel (Cat#12014634, Bio-Rad Laboratories, Hercules, CA, USA) and measured in the Luminex (Bio-Rad Bio-Plex 200) system according to the manufacturer’s instructions. The serum samples were diluted 1:100 into the Bio-Plex sample buffer, and 50 μl of the dilution was pipetted onto the plate. The observed target concentrations were calculated with the help of seven threefold diluted Virotrol SARS-CoV-2 standards (Cat #200300A, Bio-Rad, Marnes-la-Coquette, France). The concentration ranges for all the targets were 5.4–4000 U/ml. Wells containing only buffer were used as a negative control.

### 2.6 RNA extraction

The PAX blood tubes were thawed and tempered at +23°C for 2h, followed by 10-time inversion of the tube to mix well. Total RNA was extracted from the PAX tubes according to the instructions in the Preserved Blood RNA Purification Kit II (for use with PAXgene™ Blood RNA Tubes) (Product #43500, Norgen Thorol, ON, Canada). Extraction was done according to the manufacturer’s instructions. Briefly, the PAXgene™ Blood RNA tubes were centrifuged for 10 min at 4,000 × g using a swing bucket rotor. The supernatants were discarded, and 4 mL of NPX1 buffer was added to the pellets, which were vortexed and pipetted up and down to enhance pellet redissolving. The tubes were again centrifuged for 10 min at 4,000 × g, and supernatants were discarded. Then 600 μL of NPX2 buffer was added to the pellet, dissolved by vortexing, and the lysate was centrifuged at 14,000 × g for 2 min to remove any insoluble materials. This centrifugation was repeated when needed. The cleared supernatant was mixed with an equal amount of 100% ethanol, mixed, and transferred to a column. After centrifugation for 1 min at 3,500 × g, flowthrough was discarded, and the column was washed by applying 400 μL of NPX3 to the column and centrifuged for 1 min. DNaseI treatment was done on-column in 100 μL of NPX4 buffer. After incubation at +28°C for 15 min, 400 μL of NPX4 buffer was added, and the column was centrifuged at 14,000 × g for 1 min. Wash the column again with 400 μL of NPX4 buffer and dry the column by centrifuging for 2 min. Elute RNA by adding 50 μL of NPX5 buffer and centrifuging for 2 min at 200 × g, followed by 1 min at 14,000 × g. The quantity and quality of the purified RNA were studied by Qubit and TapeStation, respectively, and the samples were stored at –80°C until used.

### 2.7 RNA-seq

RNA sequencing was performed as mRNA sequencing via Illumina platforms, based on the mechanism of SBS (sequencing by synthesis) following the standard procedures of Novogene (Durham, NC, United States). Conversion Software (bcl2fastq2) was used to convert BCL files to FASTQ file format and demultiplex the samples. Sequenced reads were trimmed for adaptor sequence and masked for low-complexity or low-quality sequence using Trimmomatic (parameters: LEADING:3, TRAILING:3, SLIDINGWINDOW:4:15, and MINLEN:36). Trimmed reads were mapped to the GRCm38.p6 whole genome using the STAR aligner (2.6.0c). Counts per gene were calculated using featureCounts software (v1.6.4). The count data and associated metadata are provided as a **Supplementary Data Sheet 1** (Count_metadata.zip).

### 2.8 Gene expression and pathway enrichment analyses

TMM normalization of raw read counts followed by differential expression analysis was performed using EdgeR and LIMMA in the R-based Express Analyst software (18, 19). The *p*-values were adjusted for multiple hypotheses testing using the Benjamini-Hochberg (B-H) procedure. To evaluate the distribution of differentially expressed genes (DEGs) between specified contrast sets, UpsetPlots were created using ExpressAnalyst. Enrichment analysis of DEGs was conducted with Enrichr (20).

### 2.9 Statistical analyses

In **Tables 1**–**3**, the clinical parameters were compared using Kruskal–Wallis tests for continuous variables and chi-squared tests for categorical variables. In **Table 3**, pairwise tests between groups were also conducted. This was done with the R statistical computing environment version 4.4.0 (21). The statistical significance analyses of differences in gene expression or SARS-CoV-2 antibody amounts were performed with GraphPad Prism 10 Software (GraphPad Software Inc., San Diego, CA). Results are expressed as mean ± SEM and *p*-values of < 0.05 were considered to be statistically significant.

**Table 1:** Demographic information of the patients.

Demographic information	PCC	Recovered	Healthy	*p*-value
Total *N* (%)	54 (31)	57 (33)	63 (36)	0.7
Female, *N* (%)	39 (72.2)	42 (73.7)	48 (76.2)	0.9
Age (years) Mean (*SD*)	44.7 (11.1)	43.9 (9.3)	44.6 (12.4)	0.9
BMI (kg/m^2^) Mean (*SD*)	27.4 (5.2)	26.7 (4.9)	26.7 (5)	0.8
At least one comorbidity *N* (%)	31 (58.5)	18 (31.6)	12 (24)	<.001
Number of comorbidities Mean (*SD*)	1.3 (1.4)	0.4 (0.7)	0.3 (0.6)	<.001
Monthly doses of alcohol Mean (*SD*)	7 (10.5)	8.9 (11.9)	7.5 (7.9)	0.3
Smoking, *N* (%)	2 (3.8)	3 (5.5)	5 (8.1)	0.7

**Table 2:** COVID-19 infection, symptoms, and functional tests during post-COVID condition.

Clinical information on COVID-19 infection and post covid 19 condition	PCC	Recovered	Healthy	*p*-value
S-CV19N-Ab positive	16 (32%)	18 (32.7%)	13 (21.3%)	0.3
Confirmed hospitalization during acute COVID-19 *N* (%)	0 (0)	0 (0)	0 (0)	–
PCC symptom duration (years) Mean (*SD*)	0.9 (0.5)	NaN (NA)	NaN (NA)	–
Time between sampling and positive PCR test (days) Mean (*SD*)	363.6 (146.6)	337.6 (129.5)	NaN (NA)	0.7
Dominant hand grip strength (kg) Mean (*SD*)	35 (7.5)	35 (9.7)	33.1 (10.2)	0.08
6MWT distance (m) Mean (*SD*)	396.2 (82.7)	647.6 (75.4)	607.2 (59.2)	<.001
Weekly rigorous exercise *N* (%)	5 (19.2)	43 (76.8)	41 (66.1)	<.001
0–10 Functional ability Mean (*SD*)	4.5 (1.9)	8.9 (0.9)	8.7 (1.3)	<.001
0–10 Quality of life Mean (*SD*)	5.1 (1.8)	8.8 (0.9)	8.5 (1.6)	<.001

**Table 3:** Comorbidities and selected clinical laboratory results of male patients.

	Control (Ctrl+C)	High-erythrocyte fraction (HighE)	Normal fraction of erythrocytes (NormE)	*p*-value
Total *N*	30	8	7	NA
Age (years) Mean (*SD*)	45.4 (12.4)	45.4 (11.5)	44.4 (10.9)	0.9
BMI (kg/m2) Mean (*SD*)	28.0 (3.9)	30.2 (5.5)	23.7 (3.5)	0.02**
B -Hb Mean (*SD*)	149.5 (9.9)	155.8 (12.2)	144.9 (8.9)	0.2
P -K Mean (*SD*)	4 (0.2)	3.6 (0.4)	3.7 (0.2)	0.01*
SP -Ca-Ion Mean (*SD*)	1.19 (0.025)	1.22 (0.029)	1.23 (0.026)	0.02*
P -APTT Mean (*SD*)	27.6 (1.9)	31.4 (1.9)	29.7 (3.4)	0.03*
P -Fibr Mean (*SD*)	2.7 (0.5)	3.2 (0.2)	2.7 (0.6)	0.05*
Elevated P -CRP *N* (%)	1 (6.7)	0 (0)	0 (0)	>.99
No of comorbidities Mean (*SD*)	0.27 (0.6)	1.5 (1.8)	1.6 (1.1)	<.001*
Smoking, *N* (%)	3 (10)	1 (12.5)	0 (0)	0.9
Asthma *N* (%)	2 (6.7)	3 (37.5)	1 (14.3)	0.09*
Diabetes *N* (%)	0 (0)	2 (25)	1 (14.3)	0.04*
Sleep Apnea *N* (%)	2 (6.7)	2 (25)	2 (28.6)	0.1
Month 0 No of symptoms Mean (*SD*)	4.4 (4.3)	23 (4.5)	19.6 (6.9)	<.001*

**Significant difference between Normal and High erythrocyte groups.

*Significant difference between PCC and control groups.

## 3 Results

### 3.1 Study population and clinical findings

Participants in the study included 54 patients suffering from PCC, 57 patients who recovered normally after COVID-19 infection, and 63 healthy controls. Patients in the PCC and Recov cohorts were recruited approximately 12 and 11 months after their positive SARS-CoV-2 nucleic acid amplification test, respectively (**Table 2**). Compared to recovered COVID-19 patients or controls, patients with PCC more often had comorbidities (58% vs. 32% vs. 24%; *p* = .05) and more frequent comorbidities (1.3 vs. 0.4 vs. 0.3, *p* <.05) (**Table 1**). They more often had nervous system disorders (28% vs. 9% vs. 10%, *p* <.05), circulatory disorders (19% vs. 5% vs. 4%, *p* <.05), respiratory disorders (32% vs. 16% vs. 10%, *p* <.05), and musculoskeletal diseases (30% vs. 14% vs. 10%, *p* <.05). Asthma was more common among the patients with PCC than among the other groups (26.4% vs. 3.5% vs. 2.0%, *p* <.05) (**Supplementary Table S1**).

None of the patients were hospitalized during the acute COVID-19 infection (**Table 2**). The patients with PCC had statistically significantly decreased functioning (self-reported) according to the functional ability test, lower quality of life, and 6MWT distance. Fewer were able to do rigorous exercise.

Of the Recov and Ctrl groups, 33% and 21% had antibodies to SARS-CoV-2 nucleocapsid protein, showing that at least one fifth of the non-COVID group had eventually also had asymptomatic COVID-19 (**Table 2**). These antibodies do not necessarily persist long after infection, and therefore, not all individuals in the PCC or recovered groups had them. Due to this, we combined the groups Recov and Ctrl as a control group for some of the analyses (**Table 3**).

### 3.2 SARS-Cov-2 antibodies

**Figure 2:**
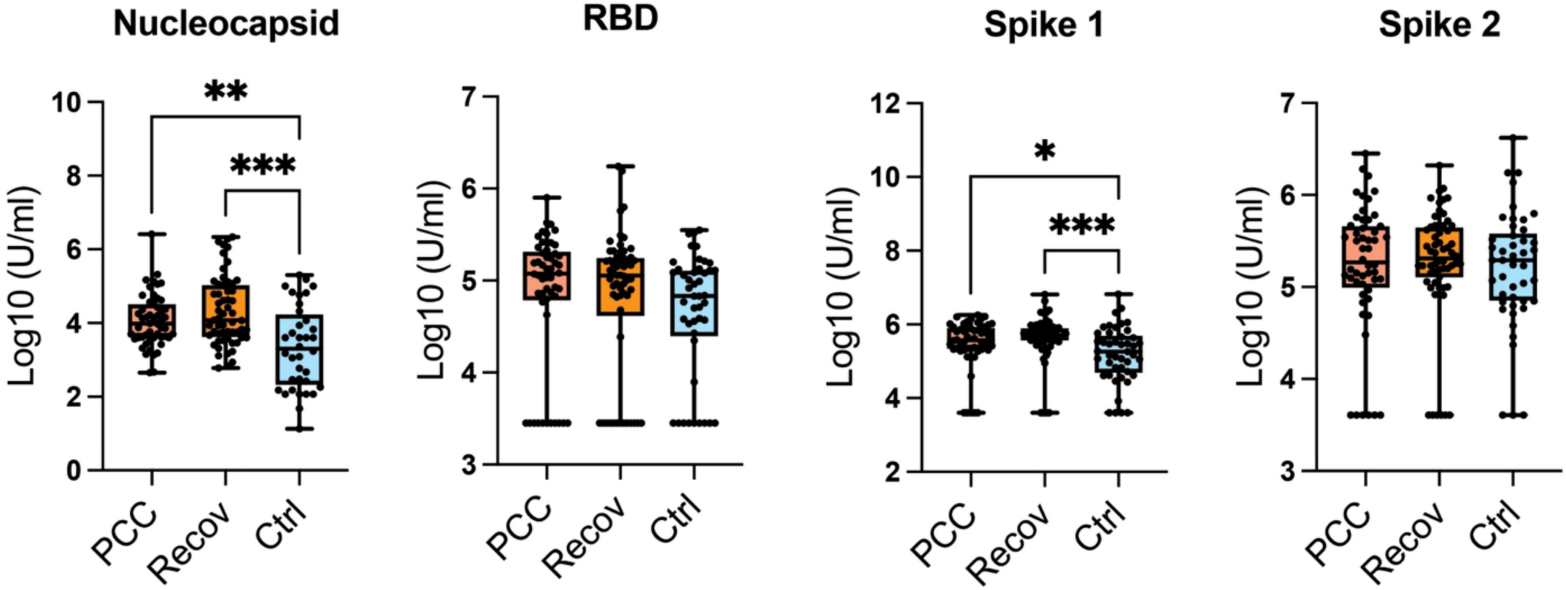
SARS-Cov-2 antibodies. The antibody concentrations for nucleocapsid, receptor binding domain (RBD), and spike proteins 1-2. RBD, spike 1 (S1), and spike 2 (S2) antigens are viral antigens in nationally provided vaccines, whereas nucleocapsid identify individuals with an immune response against naturally acquired infection. The sample sizes per study group for the antibody analysis are: PCC (*N* = 52), Recover (*N* = 57), and Ctrl (*N* = 45). RBD, spike 1 (S1), and spike 2 statistical differences were calculated with the help of the Kruskal–Wallis test with Dunn’s multiple comparison test. *p < 0.05; **p < 0.01; ***p < 0.001.

The antibody concentrations for RBD and spike proteins 2 showed no statistical significance, but their mean values in control patients are lower than in Recov or PCC groups (**Figure 2**). The antibody concentrations to nucleocapsid and spike 1 proteins were lower in the control group when compared to either of the recovered or PCC patients (**Figure 2**).

### 3.3 Transcriptome changes between PCC, Recov, and controls

**Figure 3:**
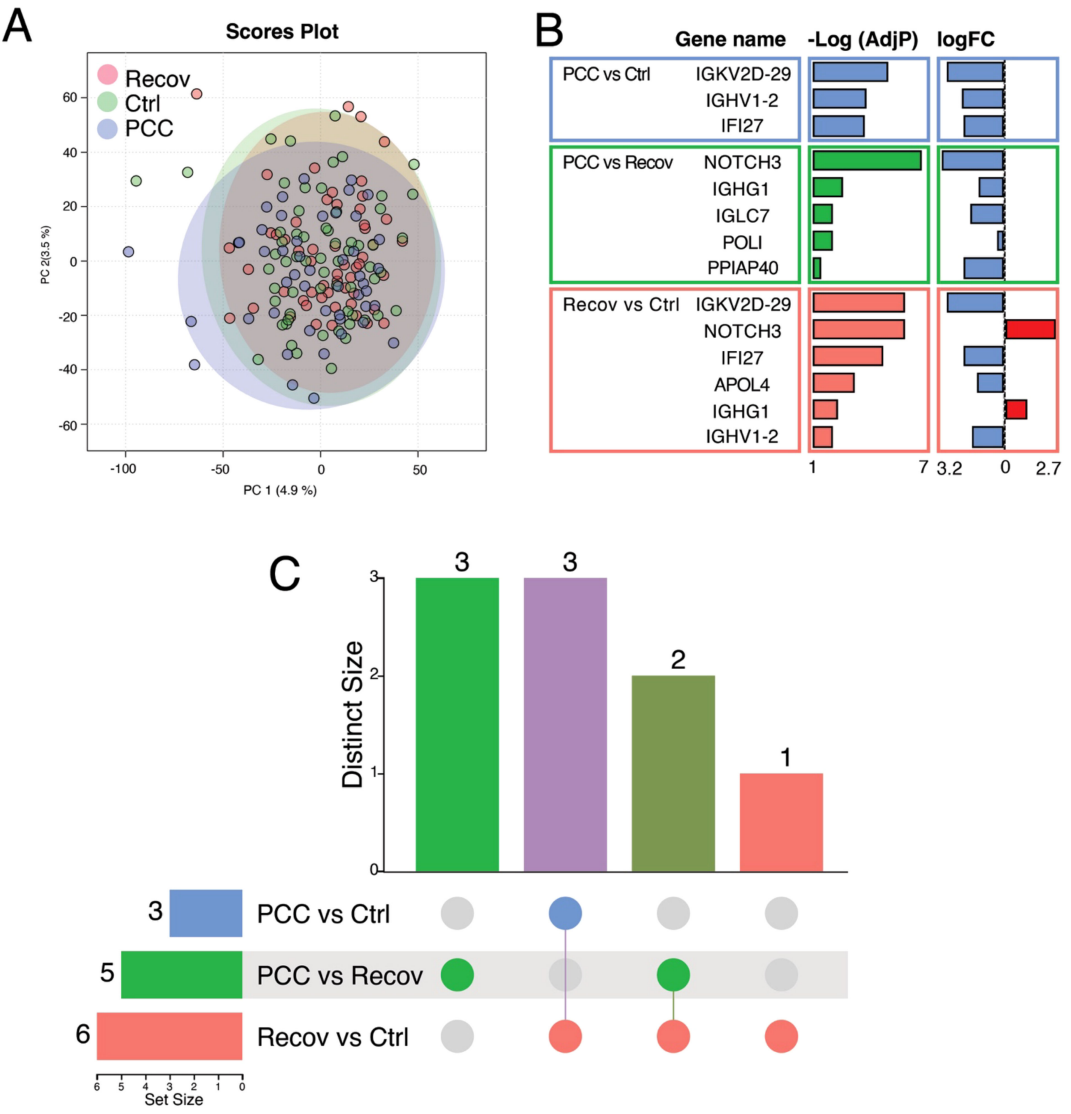
Differentially expressed genes (DEGs) between recovered covid patients (Recov) and patients with post-COVID-19 condition (PCC) or these groups when compared to healthy controls (Ctrl). **(A)** Principal component analysis of the patient groups. **(B)** The 14 DEGs after EdgeR analysis with associated negative logarithm of adjusted *P*-value [-Log(AdjP)] and logarithm of the fold change (logFC) of the gene expression when compared to Ctrl/Recov. The positive logFC is shown by red and the negative logFC is shown by blue. **(C)** Number of specific and shared DEGs between different groups. IGKV2D29, IGHV1-2 and IFI27 were shared between PCC versus Ctrl and PCC versus Recov, whereas three genes (IGLC7, POLI and PPIAP40) were specific for PCC versus Recov. The two genes, NOTCH3 and IGHG1, were specific for PCC versus Recov and Recov versus Ctrl, and APOL4 was the only specific gene for Recov versus Ctrl. In **(B, C)**, PCC versus Ctrl is indicated with blue color, PCC versus Recov by green and Recov versus Ctrl by red.

Based on the global gene expression profiles, there was a considerable overlap between the different study groups in the principal component analysis (PCA) (**Figure 3A**). The PCC group was separated from the controls (Ctrl) by three DEGs and from the recovered patients (Recov) by five DEGs, respectively (**Figure 3B**). The Recov patients were separated from the Ctrl by six DEGs (**Figure 3B**). Despite the low number of DEGs, pathway enrichment analysis in the Reactome database revealed significant alterations in immune-related pathways in PCC patients. Notably, the “Classical Antibody-Mediated Complement Activation” pathway (adj. *p* = 0.00271) was enriched in PCC versus Recov comparisons, driven by the differential expression of IGHG1 and IGLC7. Comparison of PCC versus Recov had three specific genes (IGLC7, POLI, and PPIAP40), while expression of NOTCH3 and IGHG1 genes was downregulated in PCC/Ctrl and upregulated in Recov/Ctrl (**Figures 3B, C**). The comparison of Recov versus Ctrl showed exclusive downregulation of APOL4 and shared downregulation with all of the PCC/Ctrl genes (IGKV2D-29, IGHV1-2, and IFI27) (**Figure 3C**).

### 3.4 Gender specific changes in transcriptome

When analyzing the subgroup of females only, PCC versus Ctrl had five DEGs and Recov versus Ctrl had 10 DEGs, whereas PCC versus Recov had only one DEG (NOTCH3) (**Supplementary Figure S1A**). In the PCC versus Ctrl and Recov versus Ctrl contrasts, the pathway “Immunoregulatory Interactions Between a Lymphoid and a Non-Lymphoid Cell” was significantly enriched (adj. *p*-values = 0.0006323 and 0.0001475, respectively) in the Reactome database, indicating sex-specific immune alterations. The NOTCH3 gene was also differentially expressed in the comparison of Recov/Ctrl, which additionally shared five DEGs with PCC/Ctrl (**Supplementary Figure S1B**). The top 30 DEGs did not form clear up- or downregulated gene clusters in the clustergram (**Supplementary Figure S1C**).

**Figure 4:**
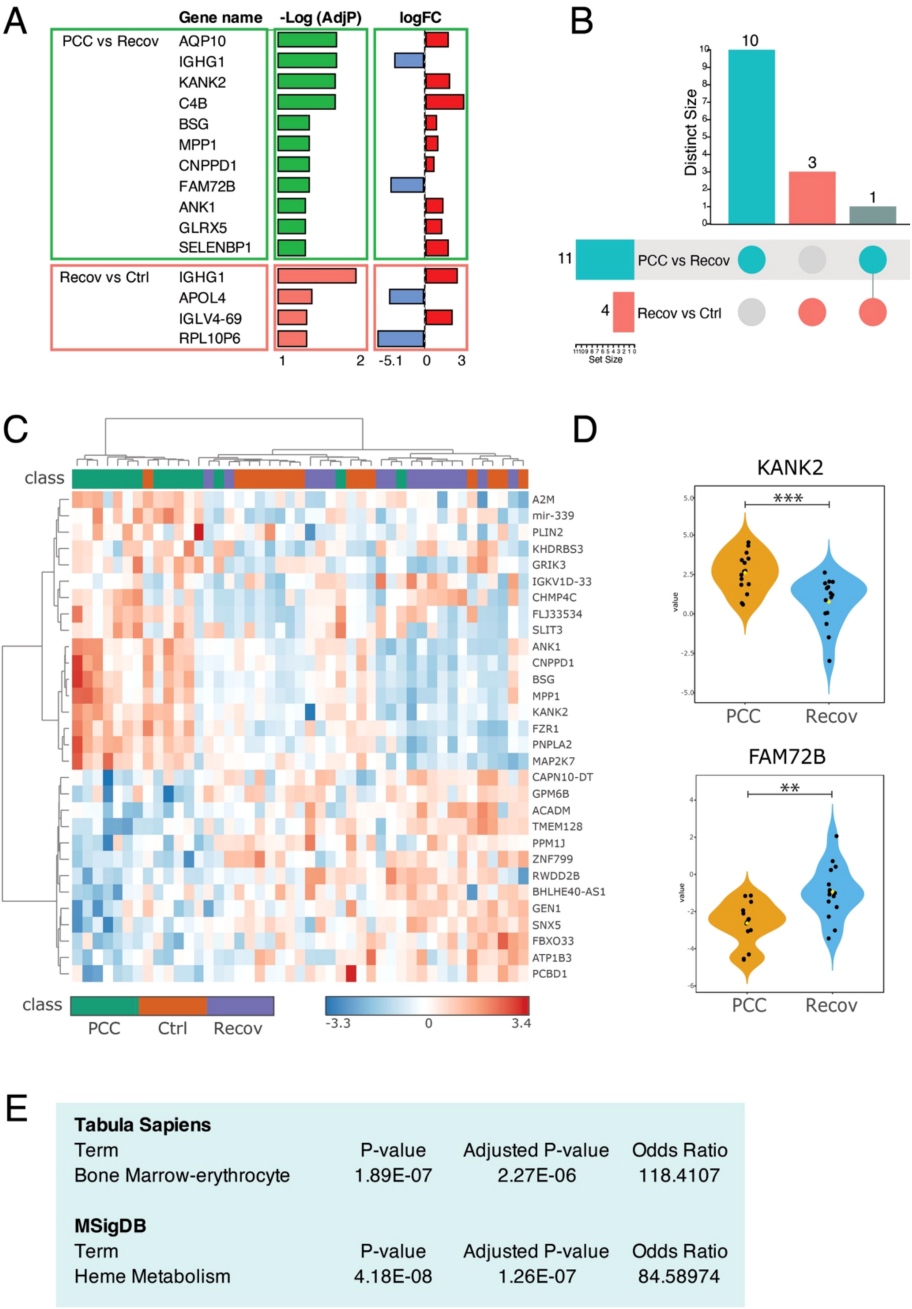
Differentially expressed genes (DEGs) between recovered male COVID patients (Recov) and patients with post-COVID-19 condition (PCC) when compared to healthy controls (Ctrl). **(A)** The 15 DEGs after EdgeR analysis. **(B)** Number of specific and shared DEGs between different groups. **(C)** The most significant 30 ANOVA-analyzed genes in the heatmap. **(D)** Example gene expressions. **(E)** Enrichment of 11 DEGs in PCC versus Recov contrast in Enrichr. **p < 0.01; ***p < 0.001.

Among the male subjects, patients with PCC had 11 DEGs when compared to Recov patients, which differed from the controls by four genes (**Figure 4A**). All 11, except the IGHG1 gene, were specific to the PCC/Recov comparison (**Figure 4B**). In the clustergram, the upregulated and downregulated gene expressions drove the formation of a cluster of patients with PCC, while Recov and Ctrl patients were inseparable (**Figure 4C**). The gene expressional differences were significant between PCC and Recov groups, as shown by the KANK2 and FAM72B genes (**Figure 4D**). In the pathway analyses, the 11 PCC-specific DEGs showed highly significant enrichment to “Bone Marrow-erythrocyte” in the Tabula Sapiens database and to “Heme metabolism” in the MSigDB database (**Figure 4E**).

### 3.5 Subset of male patients with PCC demonstrate a high fraction of erythroblasts and have abnormal heme metabolism and biosynthesis in blood transcriptome

**Figure 5:**
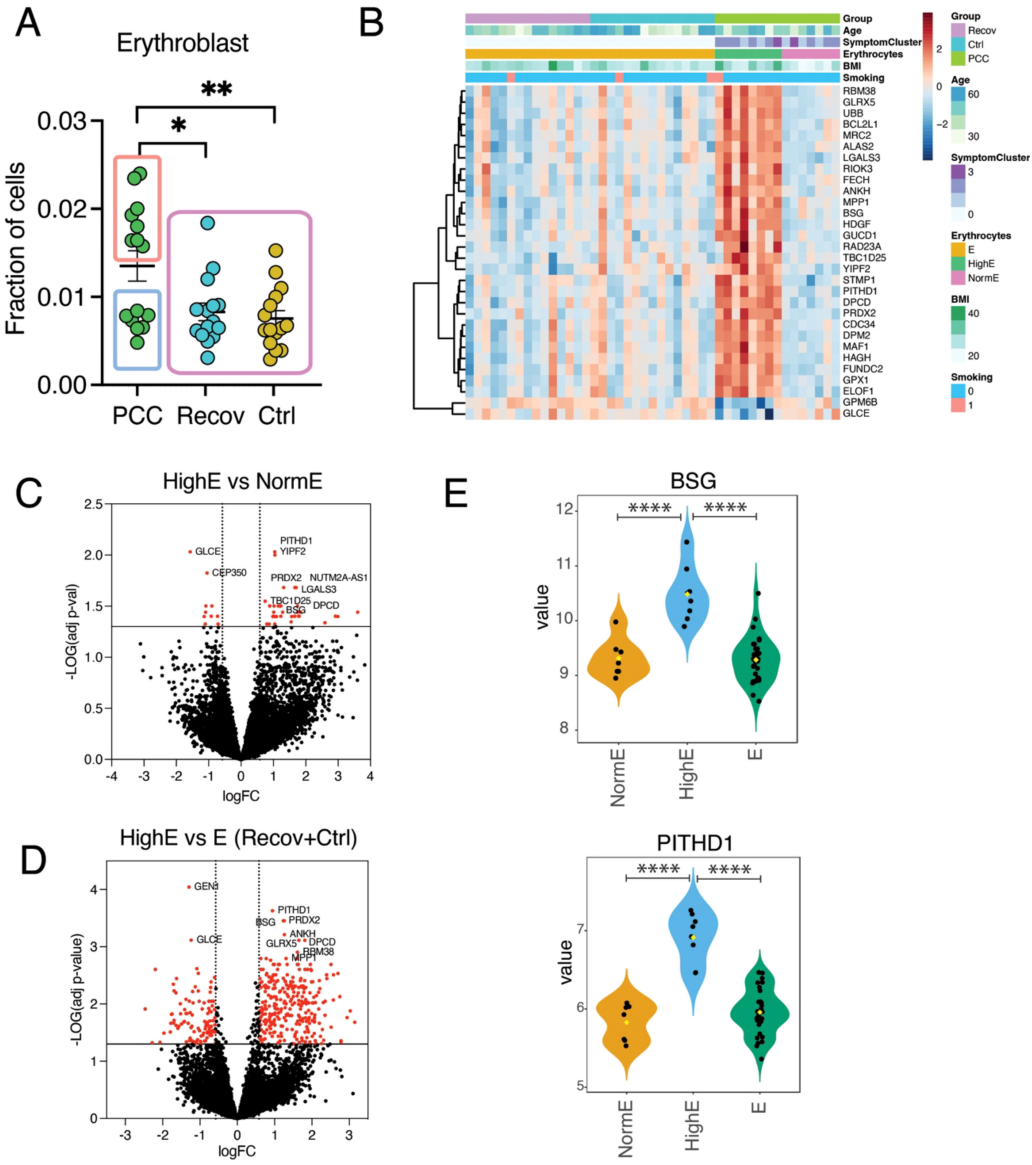
Erythroblasts play a role in male post-COVID-19 condition (PCC) patients. **(A)** Deconvoluted fractions of the erythroblasts of PCC male patients are increased when compared to recovered covid-patients (Recov) and healthy control (Ctrl) individuals. **(B)** The most significant 30 limma-analyzed genes in the heatmap, which describes also person age, group, cluster of symptoms, fraction of deconvoluted erythroblasts, BMI, and smoking. Volcano plots show the limma-adjusted (for age, BMI, and smoking), differentially expressed genes (DEGs) **(C)** between male normal fraction of erythrocytes (NormE) versus PCC male high-erythrocyte fractions (HighE), and **(D)** between Recov and Ctrl **(E)** versus HighE. The statistically significant genes (-LogP > |1.3|, logarithmic fold change > |0.58|) are shown in red-filled circles [45 DEGs in **(C)** and 399 DEGs in **(D)**] and top 10 genes are indicated with gene symbols. Gene expression of the limma-adjusted BSG and PITHD1 genes in different groups representing variable fraction of erythroblasts. *p < 0.05; **p < 0.01; ****p < 0.0001.

To identify the possible cellular differences between PCC, Recov, and Ctrl male subjects, we used a deconvolution method to identify transcriptional cell subtype fractions. There were no differences between PCC, Recov, and Ctrl in any of the 21 immune cell types analyzed when all the patients or female patients were included (data not shown). However, the men suffering from PCC had a statistically increased fraction of erythroblasts when compared to the Recov and Ctrl groups (**Figure 5A**). A heatmap of the top 30 genes from the limma analysis showed that PCC patients with a high erythrocyte fraction (HighE) formed a distinct gene cluster compared to those with a normal erythrocyte fraction (NormE) or to the Recov and Ctrl groups (**Figure 5B**). The males with PCC and a high fraction of erythroblasts had 45 DEGs and 399 DEGs when compared to males with PCC and a normal fraction of erythrocytes or to males in both the Recov and Ctrl groups, respectively (**Figures 5C, D**). The majority of these DEGs were upregulated in the HighE groups, as shown with the example genes BSG (Basigin) and PITHD1 (Recov-Terminal Proteasome-Interacting Domain of Thioredoxin-Like), which is involved in the positive regulation of megakaryocyte differentiation (**Figure 5E**).

### 3.6 Clinical and laboratory findings in a subset of male patients and their impact on transcriptome

To further analyze differences between the male HighE group and the male NormE group among males with PCC, we assessed various clinical background information and clinical laboratory results between the groups and compared them to a combined control group (Recov and Ctr) (**Table 3**). HighE and NormE male patients had a mean of 23 and 20 PCC symptoms, respectively, out of a list of 24 somatic and 20 mood symptoms, which were significantly more frequent than among the normally recovered and healthy controls (four symptoms; *p* <.05). However, only BMI was significantly higher (30.2 vs. 23.7) in the HighE group compared to the other two groups. Hemoglobin levels were also higher (155 vs. 145) between the HighE and NormE male groups, but this did not reach statistical significance. Among these, one of the HighE males had very high Hb (188 g/l), B-Eryt (6.27 × 10^12/l), and hematocrit (56%), necessitating further studies for erythropoietin and the JAK2 gene to rule out polycythemia vera. Three of the HighE males had asthma, one had sleep apnea, and all were obese (BMI > 25) (**Table 3**). Only two HighE male patients were previously relatively healthy except for hypertension or hypercholesterolemia. Thus, we cannot rule out if the background diseases compensatorily increased the hemoglobin concentration in these HighE males due to possible oxygen desaturation. However, one of the NormE males also had asthma, and two had sleep apnea. All were of Caucasian origin. The HighE male group also had a longer APTT and higher fibrinogen levels than the controls, but there were no differences in the C-reactive protein (CRP) level as an inflammation marker (**Table 3**).

To investigate the effect of BMI on transcriptional changes in the subset of male patients with PCC, we conducted limma analysis with and without adjustments for BMI and also for age and smoking. The use of adjustments reduced the number of DEGs from 137 to 44 in HighE versus NormE male patients with PCC (**Supplementary Figure S2A**). When comparing the males in the Recov and Ctrl groups to the group of HighE males with PCC, the number of statistically significant DEGs decreased by 88 genes (from 528 to 440 DEGs) (**Supplementary Figure S2B**). In both comparisons, the DEGs were enriched in the “Blood-erythrocyte” and “Heme metabolism” categories in the Tabula Sapiens and MSigDB databases, respectively (**Supplementary Figures S2C**, **D**). Additionally, the DEGs between HighE and samples from recovered patients and healthy control patients were enriched in “Abnormality of the heme biosynthetic pathway” in the Human Phenotype Ontology database (**Supplementary Figure S2D**).

## 4 Discussion

In this cross-sectional study on peripheral blood cell gene transcriptomics of patients with PCC, we detected few DEGs between cases and the controls, those who recovered normally and those who never had a COVID-19 infection according to their knowledge. Thus, the study did not reveal clear mechanisms that might explain the pathogenesis of PCC. However, we found DEGs in a subset of male patients with PCC. These suggested differences in gene transcription in erythroblasts and heme synthesis, but the mechanism is unclear.

The clinical parameters did not explain why patients in the PCC group had poor results in the functional tests. Among all patients with PCC, serum potassium levels were slightly lower and ionized calcium levels were slightly higher than among controls, but both were still within the normal range, making the clinical significance unclear. Some patients used corticosteroids, testosterone supplementation, and diuretics, which may affect potassium levels. Among male patients with PCC, APTT, and fibrinogen levels were slightly higher than among controls. The clinical significance of these findings remains uncertain. Although measuring lupus anticoagulant would have provided valuable insights into the increased APTT, this parameter was not assessed in our study. Additionally, despite fibrinogen being an acute phase reactant, CRP levels were not elevated.

The concentrations of total RBD- or spike-specific antibodies were slightly increased in our PCC and Recov groups compared to healthy controls, suggesting that COVID-19 infection may enhance the production of vaccine-related antibodies for an extended period. Additionally, nucleocapsid-specific antibodies were significantly higher in the PCC and Recov groups than in controls, indicating that asymptomatic infection results in a lower antibody response to the viral nucleocapsid compared to symptomatic infection. Although we did not find differences between PCC and Recov groups, Yin et al. showed significantly higher (2.3×) RBD-specific antibody titers in PCC when compared to Recov individuals (22). Additionally, they found a negative correlation, in which patients with PCC having the highest frequencies of SARS-CoV-2–specific CD8 T cells had nearly undetectable antibody levels to the RBD (22). In our studies, we did not find any PCC or Recov patients with very low anti-RBD antibodies. Additionally, there is a large inter-individual variation in SARS-COVID-19–specific antibody concentrations, which also could be explained by normal variation of an individual’s immune responses, especially an improper crosstalk between the cellular and humoral adaptive immunity during viral infection. However, virus-specific antibody measurements stay a valid tool for diagnostics.

We found only very modest changes in the peripheral blood mononuclear cell (PBMC) transcriptome between all recovered COVID-19 patients or current patients with PCC and healthy controls. However, both PCC and Recov patients showed significant changes in the expression of immunoglobulin (Ig) light or heavy chain variable genes (IGKV2D-29 and IGHV1-2), which play a role in the antigen-binding activity of antibodies and B cell receptors when compared to healthy controls. When we compared PCC or Recov females to control females, a few more immunoglobulin genes were found to be significantly different (IGHV3-53, IGKV6-21, and IGLV2-18). Others have shown that infection severity and virus variants prefer expression of different regions of Ig VDJ genes (23). Severe SARS-CoV-2 infection is shown to be associated with unique B-cell signatures and enrichment of the variable region of VH3-53, which is also described to be the dominant neutralizing antibody in response against SARS-CoV-2 (23). Between PCC and Recov groups, we found only five DEGs in the whole study population and only one DEG in females. These genes included additional immunoglobulin genes (IGHG1 and IGLC7) and NOTCH3, which is a signaling receptor typically expressed by endothelial cells and which is shown to be dysregulated after COVID-19 infection (24). Others have also reported only minor transcriptomic changes between different PCC versus Recov study populations. Yin et al. identified only two DEGs (OR7D2, a G-protein–coupled receptor, and ALAS2, an enzyme catalyzing heme synthesis) that were significantly overexpressed in the RSEQ analysis of the whole blood between PCC and Recov groups (22). Similarly, An et al., found only two DEGs (GYPE, glycophorin E, and ALDH1A1, aldehyde dehydrogenase 1 family member A1) between PCC and convalescent Recov patients (25). The low number of DEGs suggests that transcriptomic changes associated with PCC may be influenced by individual variability, including genetic background, comorbidities, microbiome composition, diet, and treatments. Alternatively, PBMC transcriptomic alterations in PCC might be minimal or transient. Despite this limitation, pathway enrichment analysis highlights the role of immune dysregulation in PCC. Specifically, the enrichment of “Classical Antibody-Mediated Complement Activation” suggests that antibody-mediated immune responses may contribute to PCC symptom persistence. Additionally, the statistically significant enrichment of “Immunoregulatory Interactions” in females may indicate potential sex-specific differences in PCC pathophysiology. These findings align with previous reports suggesting long-term immune modulation following SARS-CoV-2 infection.

In contrast to females, the gene expression of male patients with PCC differed from Recov male patients and was enriched in erythrocytes and heme metabolism. Interestingly, about half of the male patients with PCC showed an enhanced predicted fraction of erythroblasts in cellular deconvolution. Consistent with this, males with PCC and with a high fraction of erythroblasts demonstrated 45 DEGs and 399 DEGs when compared to males with PCC and with a normal fraction of erythrocytes and to males in both the Recov and Ctrl groups, respectively. These DEGs showed high enrichment in genes related to erythrocyte development and differentiation. It is also noteworthy that this subgroup exhibited a higher BMI compared to male patients with PCC and with a normal fraction of erythroblasts. It is well known that high BMI is a risk factor for PCC (26), and our results suggest it may also directly or indirectly contribute to erythrocyte function in a subgroup of male patients with PCC. Ryad et al. (27) followed the transcriptome of PBMCs in 69 patients recovering from mild, moderate, severe, or critical COVID-19 in comparison to healthy uninfected controls at 12, 16, and 24 weeks post-infection (wpi). Of these patients, 21 were referred to a long COVID clinic, and > 50% reported ongoing symptoms more than 6 months post-infection. Only at 24 wpi, but not at 12 or 16 wpi, there were 446 DEGs when patients with PCC were compared to convalescent individuals. The genes showed very strong enrichment for platelet-related pathways that was associated with a strong downregulation of platelet and megakaryocyte gene sets among individuals. The authors suggest that changes to the blood transcriptome persist in PCC individuals, whereas they tend to resolve in convalescent individuals (27). Altogether, we can conclude that changes in genes playing a role in the regulation and function of megakaryocytes, platelets, or erythrocytes appear at least in a certain group of patients depending on the cohort. Causality remains an open issue, as we cannot rule out a confounding effect of underlying diseases.

Several problems with the human red blood cells have been reported during the acute COVID-19 disease and the during chronic period of PCC symptoms. Human red blood cell (RBC) precursors express the ACE2 receptor and CD147 (28), which makes them susceptible to SARS-CoV-2 infection and leads to impairment of hemoglobin homeostasis and aggravation of acute COVID-19 disease (29). Shen et al. (30) followed 11 severe and 35 non-severe COVID-19 patients and showed that the number of erythrocytes and platelets varied during the COVID-19 disease and recovery period, being the lowest at the discharge and then slowly increasing over time (30). Later, Kronstein-Wiedemann et al. showed that patients with PCC had impaired erythrocyte functionality (31). Although inflammation and low iron levels in blood are both a natural part of the immune response to infection, they contribute to anemia, iron dysregulation, and disruption of healthy red blood cell production, which can be seen as early as two weeks post COVID-19 in those individuals reporting PCC many months later independent of age, sex, or initial COVID-19 severity (32). Therefore, Hanson et al. suggested that problems with iron levels and the body’s ability to regulate it due to SARS-CoV-2 infection might contribute to triggering PCC (32).

A key strength of our study is the inclusion of age-matched controls recruited from the same geographical location and during the same period as the Recov and PCC cases. This ensured that both cases and controls had similar exposures to pandemic-related public infection control measures, disrupted social services, and psychosocial stress. To account for potential confounders, we conducted a comprehensive assessment of comorbidities across study groups during the 11–12 months post-infection period. The distinct erythrocyte-related transcriptomic alterations observed in a subset of male PCC patients suggest a condition-specific effect rather than incidental findings. While external factors cannot be entirely ruled out, our analysis reduces the likelihood of major confounding effects.

Our findings indicate that mild SARS-CoV-2 infection causes complex PCC, which is a heterogenous and multifactorial disease that might be better explained by the prevailing biopsychosocial factors, such as pain and stress responses (33). However, the small sample size, particularly among male patients, is a limitation in our current study. Due to this, we cannot conclusively determine if the observed transcriptomic changes have a direct biological impact on PCC pathogenesis in males. Further studies with larger cohorts of male patients, along with mechanistic studies focused on blood erythrocytes, are needed to validate the clinical relevance of our findings.

In conclusion, the past COVID-19 illness or current symptoms of PCC caused only very slight changes in the blood cell transcriptome when compared to each other or to the controls. However, a subgroup of male patients with PCC had an increased fraction of erythroblasts and enrichment of erythrocyte functions in the transcriptome, suggesting a compensatory mechanism to overcome the effects of low iron concentration or dysfunctional erythroblasts. This highlights the importance of further investigating this subgroup, as their specific responses in erythrocytes may offer valuable biomarkers for diagnosis and therapeutics. Nevertheless, the clinical relevance of these findings needs to be confirmed in larger studies.

## Data Availability

The original contributions presented in this study are publicly available. This data can be found here: https://doi.org/10.6084/m9.figshare.28525220.v1. Due to GDPR regulations and the data protection policies of Helsinki University Central Hospital (HUCH), additional individual-level metadata, including pseudonymized data, cannot be publicly shared. However, we are committed to transparency and reproducibility. Access to key aggregated clinical parameters may be provided on a case-by-case basis, subject to approval and compliance with data transfer agreements. For research inquiries: Omics data: Prof. Harri Alenius (Harri.Alenius@helsinki.fi), Clinical data: Dr. Helena Liira (Helena.Liira@hus.fi).

## Supplementary material

Supplementary Figure 1 Differentially expressed genes (DEGs) between recovered female COVID patients (Recov) and patients with post-COVID-19 condition (PCC) or these groups when compared to healthy controls (Ctrl). **(A)** The 16 DEGs after EdgeR analysis. **(B)** Number of specific and shared DEGs between different groups. **(C)** The most significant 30 limma-analyzed genes in the heatmap, which describes also person age, group and cluster of symptoms.

Supplementary Figure 2 Adjustment for age, BMI, or smoking does not eliminate the gene expression differences in high-erythrocyte male patients. **(A)** PCC males with normal fraction of erythrocytes (NormE) versus PCC males with high-erythrocyte fractions (HighE), and **(B)** between HighE versus erythrocyte fractions in Recov + Ctrl groups **(E)**. Enrichment of statistically significant genes with and without adjustment (blue and green symbols) in A are shown in **(C)** and statistically significant genes with and without adjustment (blue and green symbols) in **(B)** are shown in **(D)**.
