# Supplementary figures and images for: Patients with post-COVID-19 condition show minor blood transcriptomic changes, with altered erythrocyte gene expression in a male subgroup
Source: Front Immunol. 2025 Mar 21;16:1500997. doi: 10.3389/fimmu.2025.1500997 (PMC11968430; doi:10.3389/fimmu.2025.1500997)

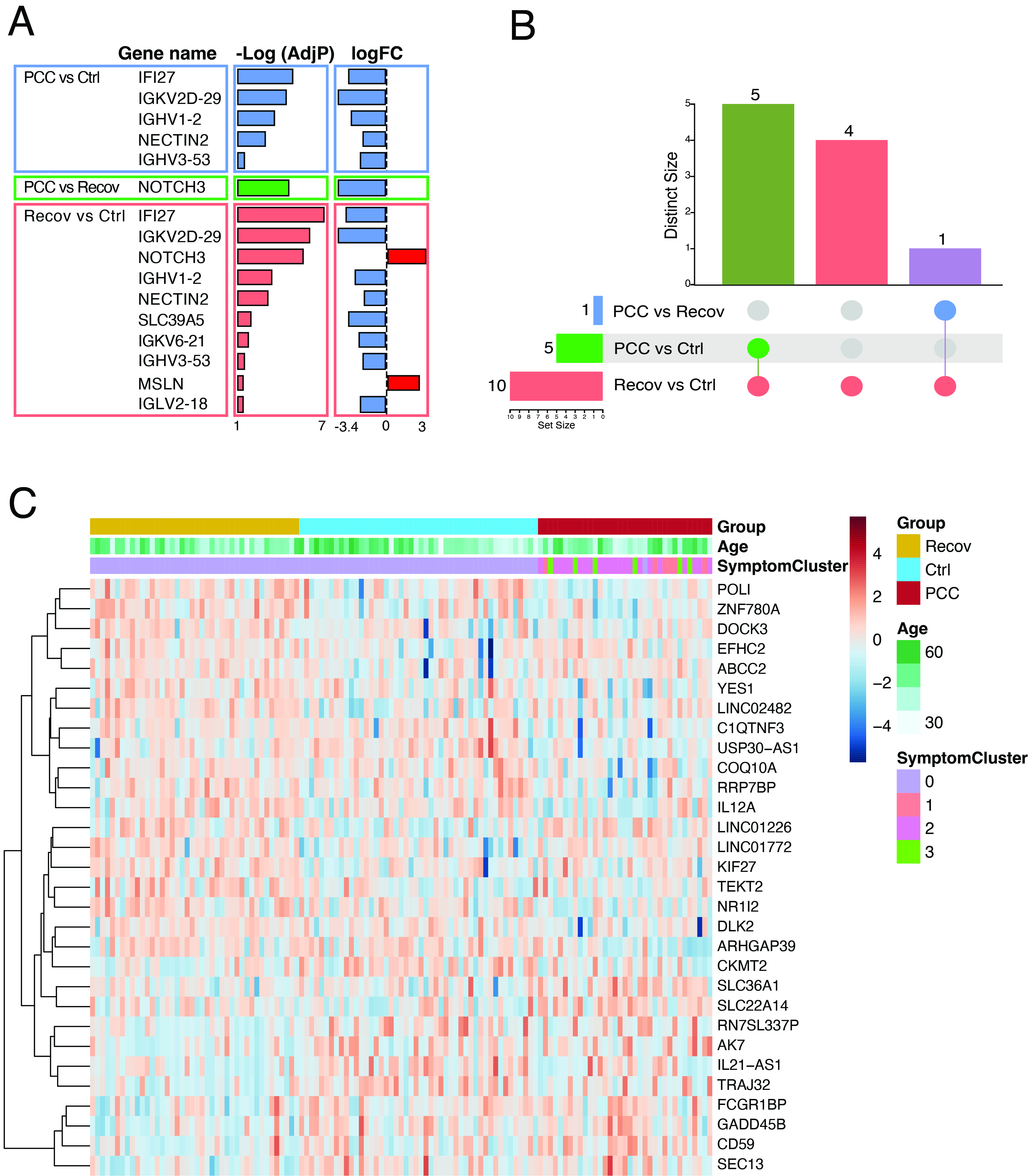

Supplement: Supplementary Figure 1 — Differentially expressed genes (DEGs) between recovered female COVID patients (Recov) and patients with post-COVID-19 condition (PCC) or these groups when compared to healthy controls (Ctrl). (A) The 16 DEGs after EdgeR analysis. (B) Number of specific and shared DEGs between different groups. (C) The most significant 30 limma-analyzed genes in the heatmap, which describes also person age, group and cluster of symptoms. [file Image1.jpg]

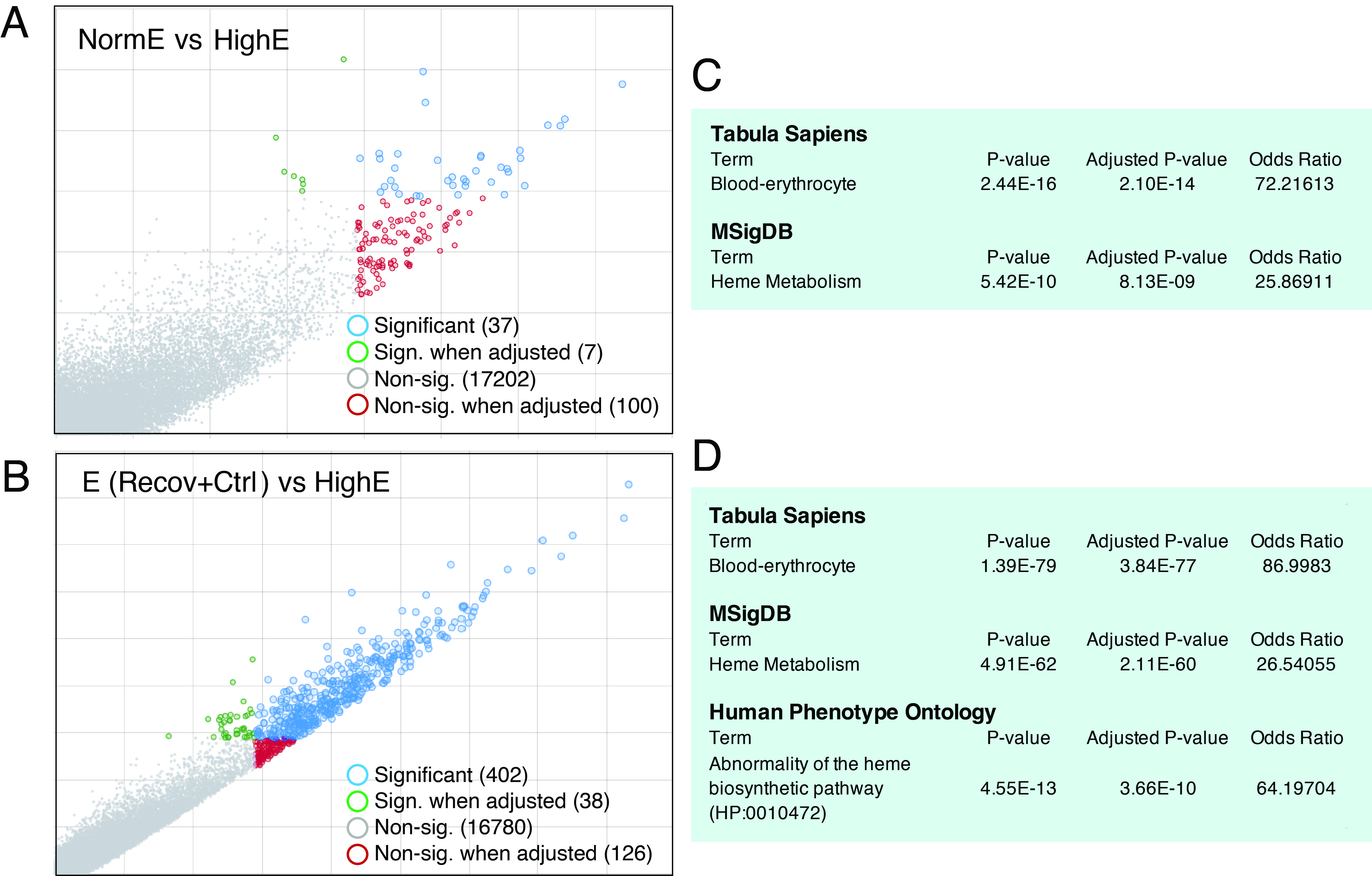

Supplement: Supplementary Figure 2 — Adjustment for age, BMI, or smoking does not eliminate the gene expression differences in high-erythrocyte male patients. (A) PCC males with normal fraction of erythrocytes (NormE) versus PCC males with high-erythrocyte fractions (HighE), and (B) between HighE versus erythrocyte fractions in Recov + Ctrl groups (E). Enrichment of statistically significant genes with and without adjustment (blue and green symbols) in A are shown in (C) and statistically significant genes with and without adjustment (blue and green symbols) in (B) are shown in (D). [file Image2.jpg]
