# Supplementary material for: Patients with post-COVID-19 condition show minor blood transcriptomic changes, with altered erythrocyte gene expression in a male subgroup
Source: Front Immunol. 2025 Mar 21;16:1500997. doi: 10.3389/fimmu.2025.1500997 (PMC11968430; doi:10.3389/fimmu.2025.1500997)
Supplement: Supplementary file 4 [file Table1.docx]

**Table S1**

| **Previous diseases, coded in tertiary care within the last 5 years** | **PCC** | **Recovered** | **Healthy** | **p-value** |
| --- | --- | --- | --- | --- |
| **Diseases class** |  |  |  |  |
| A Infectious diseases N (%) | 8(15.1) | 4(7) | 2(4) | 0.1 |
| C Neoplasms N (%) | 0(0) | 2(3.5) | 1(2) | 0.7 |
| D Blood disorders N (%) | 3(5.7) | 7(12.3) | 4(8) | 0.5 |
| E Endocrine diseases N (%) | 9(17) | 3(5.3) | 5(10) | 0.2 |
| F Mental disorders N (%) | 10(18.9) | 3(5.3) | 4(8) | 0.05 |
| G Nervous system N (%) | 15(28.3) | 5(8.8) | 5(10) | 0.01 |
| H Eye diseases N (%) | 7(13.2) | 2(3.5) | 3(6) | 0.2 |
| H Ear diseases N (%) | 4(7.5) | 1(1.8) | 4(8) | 0.3 |
| I Circulatory system N (%) | 10(18.9) | 3(5.3) | 2(4) | 0.01 |
| J Respiratory diseases N (%) | 17(32.1) | 9(15.8) | 5(10) | 0.01 |
| K Digestive diseases N (%) | 10(18.9) | 6(10.5) | 5(10) | 0.3 |
| L Skin diseases N (%) | 4(7.5) | 1(1.8) | 2(4) | 0.4 |
| M Musculoskeletal N (%) | 16(30.2) | 8(14) | 5(10) | 0.02 |
| N Genitourinary N (%) | 9(17) | 6(10.5) | 8(16) | 0.6 |
| O Pregnancy related N (%) | 6(11.3) | 3(5.3) | 7(14) | 0.3 |
| P Perinatal conditions N (%) | 0(0) | 0(0) | 0(0) | 0.8 |
| Q Congenital anomalies N (%) | 1(1.9) | 1(1.8) | 0(0) | >.99 |
| R Symptoms and signs N (%) | 29(54.7) | 9(15.8) | 6(12) | <.001 |
| S Injury and poisoning N (%) | 11(20.8) | 6(10.5) | 9(18) | 0.4 |
| V External causes N (%) | 1(1.9) | 1(1.8) | 2(4) | 0.7 |
| Z Health status N (%) | 14(26.4) | 17(29.8) | 15(30) | 0.9 |
| **Specific diagnoses** |  |  |  |  |
| Anemia N (%) | 0(0) | 0(0) | 1(2) | 0.3 |
| Asthma N (%) | 14(26.4) | 2(3.5) | 1(2) | <.001 |
| Sleep Apnea N (%) | 7(13.2) | 2(3.5) | 2(4) | 0.08 |
| Diabetes N (%) | 3(5.7) | 0(0) | 0(0) | 0.07 |
| Kidney diseases N (%) | 7(13.2) | 3(5.3) | 5(10) | 0.4 |
| Coronary Artery Disease N (%) | 1(1.9) | 0(0) | 0(0) | 0.7 |
| Cardiac Arrhythmia N (%) | 4(7.5) | 1(1.8) | 1(2) | 0.3 |
| Hypertension N (%) | 2(3.8) | 0(0) | 0(0) | 0.2 |
| Cancer N (%) | 0(0) | 2(3.5) | 1(2) | 0.7 |
| Back or Neck Disease N (%) | 7(13.2) | 3(5.3) | 3(6) | 0.3 |
| Migraine N (%) | 4(7.5) | 2(3.5) | 0(0) | 0.1 |
| Anxiety Disorder N (%) | 3(5.7) | 1(1.8) | 0(0) | 0.2 |
| Depression N (%) | 1(1.9) | 1(1.8) | 1(2) | >.99 |
